# Enzyme-enhanced RNA isolation from biofilm-producing bacteria

**DOI:** 10.1128/spectrum.03077-25

**Published:** 2026-02-27

**Authors:** Samantha M. Felton, Joseph M. Ficarrotta, Glynis L. Kolling, Jason A. Papin, Bryan W. Berger

**Affiliations:** 1 Department of Biomedical Engineering, University of Virginia 2358, Charlottesville, Virginia, USA; 2 Department of Medicine, Division of Infectious Diseases and International Health, University of Virginia 2358, Charlottesville, Virginia, USA; 3 Department of Biochemistry & Molecular Genetics, School of Medicine, University of Virginia 2358, Charlottesville, Virginia, USA; 4 Department of Chemical Engineering, University of Virginia 2358, Charlottesville, Virginia, USA; Emory University School of Medicine, Atlanta, Georgia, USA

**Keywords:** biofilms, polysaccharides, RNA isolation, *Pseudomonas aeruginosa*, enzyme, Smlt1473, *Pseudomonas syringae*

## Abstract

RNA isolation is a critical first step for gene expression analysis; however, obtaining high-quality RNA from polysaccharide-rich or biofilm-producing microbial samples remains challenging. High polysaccharide content hinders cell lysis, decreases RNA yield, and reduces sample purity, limiting the reliability and accuracy of downstream techniques such as RNA-seq and RT-qPCR. In this study, we evaluated the use of polysaccharide lyase, Smlt1473, as a pre-processing step to improve RNA isolation from *Pseudomonas* species. Incorporation of Smlt1473 into a commercial RNA extraction kit workflow significantly improved RNA extraction for mucoid clinical and agricultural pseudomonads and did not adversely affect the non-mucoid isolate, PA14. More specifically, RNA-seq analysis demonstrated that pre-processing with Smlt1473 increased the proportion of assigned reads without introducing significant changes in gene expression. Only a limited set of genes—primarily hypothetical proteins and potential phage-associated elements—were differentially expressed, while global transcriptional profiles remained stable. Together, these findings demonstrate that pre-processing with Smlt1473 provides an effective, easily integrated method to enhance RNA isolation from biofilm-forming bacteria and improves RNA-seq data quality without perturbing the underlying biology.

**IMPORTANCE:**

*Pseudomonas aeruginosa*, along with other clinically relevant pathogens, is notorious for forming complex biofilms. Microbial biofilms can be composed of anywhere from 50% to 90% polysaccharides. This high polysaccharide content of microbial biofilms severely hinders RNA extraction by complicating bacterial cell lysis, causing a decrease in yield and purity. Challenges with isolating RNA from clinically relevant biofilm-forming pathogens limit our ability to study and better understand bacterial pathogenesis. Low quality and quantity of RNA impede the accuracy and reproducibility of downstream analysis and may ultimately obstruct the discovery of novel drug targets and therapeutic interventions. Developing strategies to overcome these barriers, such as enzymatic pre-processing, is therefore critical to improving RNA recovery from biofilm-producing bacteria to enable more accurate transcriptomic studies that advance both basic science and clinical applications.

## INTRODUCTION

Extraction of high-quality and quantity RNA is imperative for accurate and reproducible results from molecular biology techniques such as RNA-seq and RT-qPCR that are commonly used for gene expression analysis (1–5). By using RNA to study gene expression profiles, disease biomarkers can be identified, as well as potential therapeutic targets (6). Clinically relevant pathogens, including *Pseudomonas aeruginosa*, *Staphylococcus aureus*, and *Klebsiella pneumoniae*, are significant biofilm formers, making it challenging to extract RNA from these sample types (7). Challenges in isolating quality RNA from these clinically relevant pathogens inhibit molecular testing capability and ultimately hinder our ability to better understand bacterial pathogenesis (5, 8, 9). High-quality RNA is not only necessary for scientific research but also in areas including infectious disease diagnostics and food safety (10–14). For example, it has been shown that RNA quality affected the diagnostic accuracy of SARS-CoV-2 infections during the 2019 pandemic (15, 16). Additionally, being able to extract high-quality RNA from a variety of food matrices is essential for the detection of food-borne pathogens such as norovirus and *Salmonella* (13, 17). Despite its importance in many applications, obtaining high-quality and quantity RNA is notably difficult. Stability issues and high susceptibility to degradation make RNA extraction quite arduous; furthermore, certain sample types pose additional purification hurdles. Specifically, polysaccharides found in various sample types, including plant, clinical (e.g., sputum), and microbial biofilms, affect RNA extraction (4, 11, 18, 19). Polysaccharides often entrap cells, lowering cell lysis efficiency and limiting the release of cell contents, which thereby affects RNA yield (4, 11, 20). Additionally, polysaccharides can co-precipitate with the RNA, resulting in contamination and thus decreased sample purity (18, 20, 21). Ultimately, polysaccharide content is a significant hindrance to isolating high-quality and quantity RNA, which is required for downstream analysis techniques ranging from gene expression to nucleic acid-based detection in clinical and industrial applications.

Prior to extraction, degradation of polysaccharides is a crucial step for obtaining high-quality and quantity RNA that is suitable for downstream analysis. There are currently many commercially available RNA isolation kits; however, they are not optimized for sample types with high polysaccharide content, such as microbial samples with significant biofilm (22). Many studies have isolated RNA from bacteria in the planktonic state, but few have successfully isolated RNA from polysaccharide-rich or biofilm-producing samples (4, 22–24). Those studies that have been able to successfully extract RNA from polysaccharide-rich samples use techniques such as phenol-chloroform extraction and the hexadecyltrimethylammonium bromide (CTAB) extraction method (22–24). Both of these methods use hazardous organic solvents posing health threats and complicating waste disposal (25, 26). Additionally, phase separation steps during these extraction techniques increase the likelihood of contaminant carryover, ultimately affecting final RNA purity (27–29). Both methods are time-intensive, and CTAB often even requires additional purification—such as column cleanup, lithium chloride precipitation, or DNase I treatment—after initial extraction (30–32). Overall, polysaccharides pose a major challenge for successful RNA isolation, and thus, methods to remove these macromolecules prior to the extraction process are needed. While methods such as phenol-chloroform and CTAB extraction do exist to aid in RNA extraction of polysaccharide-rich samples, their toxicity, labor intensiveness, and extra purification steps limit their utility in more sensitive nucleic acid detection applications.

In this study, we focus on the development of an enzymatic pre-processing step to improve RNA extraction from polysaccharide-rich *Pseudomonas* samples. Specifically, the clinically relevant pathogen *P. aeruginosa* has a known mucoid phenotype, defined by the overproduction of the exopolysaccharide alginate (33). With microbial cells encapsulated in this polysaccharide-rich biofilm matrix, it is difficult to obtain high-quality and quantity RNA after isolation using commercially available kits. Previously, it has been shown that polysaccharide lyase, Smlt1473, is able to inhibit and degrade alginate produced by mucoid *P. aeruginosa* clinical isolates (34). Here, we showcase the ability of recombinantly produced polysaccharide lyase, Smlt1473, to improve RNA extraction from *Pseudomonas* species. This study serves as a framework for enhancing the quality of RNA extracted from biofilm-rich bacteria. While this study focuses on *Pseudomonas* species, we anticipate that this method will be applicable to other biofilm-producing bacteria. Improving RNA isolation techniques for these polysaccharide-rich bacteria will allow for advancements in basic science, as well as in clinical and industrial settings.

## RESULTS

### Polysaccharide lyase, Smlt1473, improves RNA isolation from mucoid bacteria

**Fig 1:**
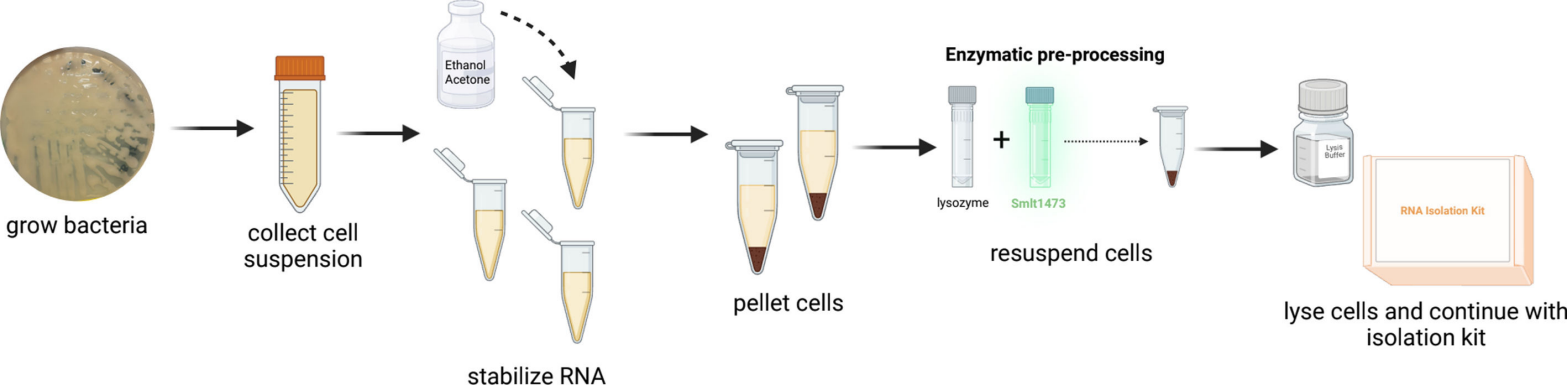
Overview of enzymatic pre-processing method. Bacteria were grown to establish the mucoid phenotype. Plate contents were collected, and RNA was stabilized. Samples were centrifuged to pellet cells, and the supernatant was discarded. Cell pellets were resuspended in 25 μL of 4 mg/mL lysozyme, in addition to 100 μg of Smlt1473. Lysis buffer was added, and the remainder of the NEB RNA isolation kit protocol was followed. Created in BioRender, Felton (2025) (https://BioRender.com/2y8rqsr).

A study by Dunphy et al., in 2021 (35), used a collection of *P. aeruginosa* clinical isolates from the University of Virginia hospital that were collected from February 2019 to February 2020. From this isolate collection, we chose one of the most robust alginate-producing clinical isolates, UVA44618 (35). Additionally, this study uses mucoid laboratory reference strain PDO300 (36). Each strain was plated from frozen stock onto LB agar plates and left to incubate at 37°C for 24 h to establish a mucoid phenotype. Plate contents were collected, and RNA was isolated in the presence and absence of 100 μg of Smlt1473, followed by use of a standard RNA isolation kit protocol from NEB (Fig. 1).

**Fig 2:**
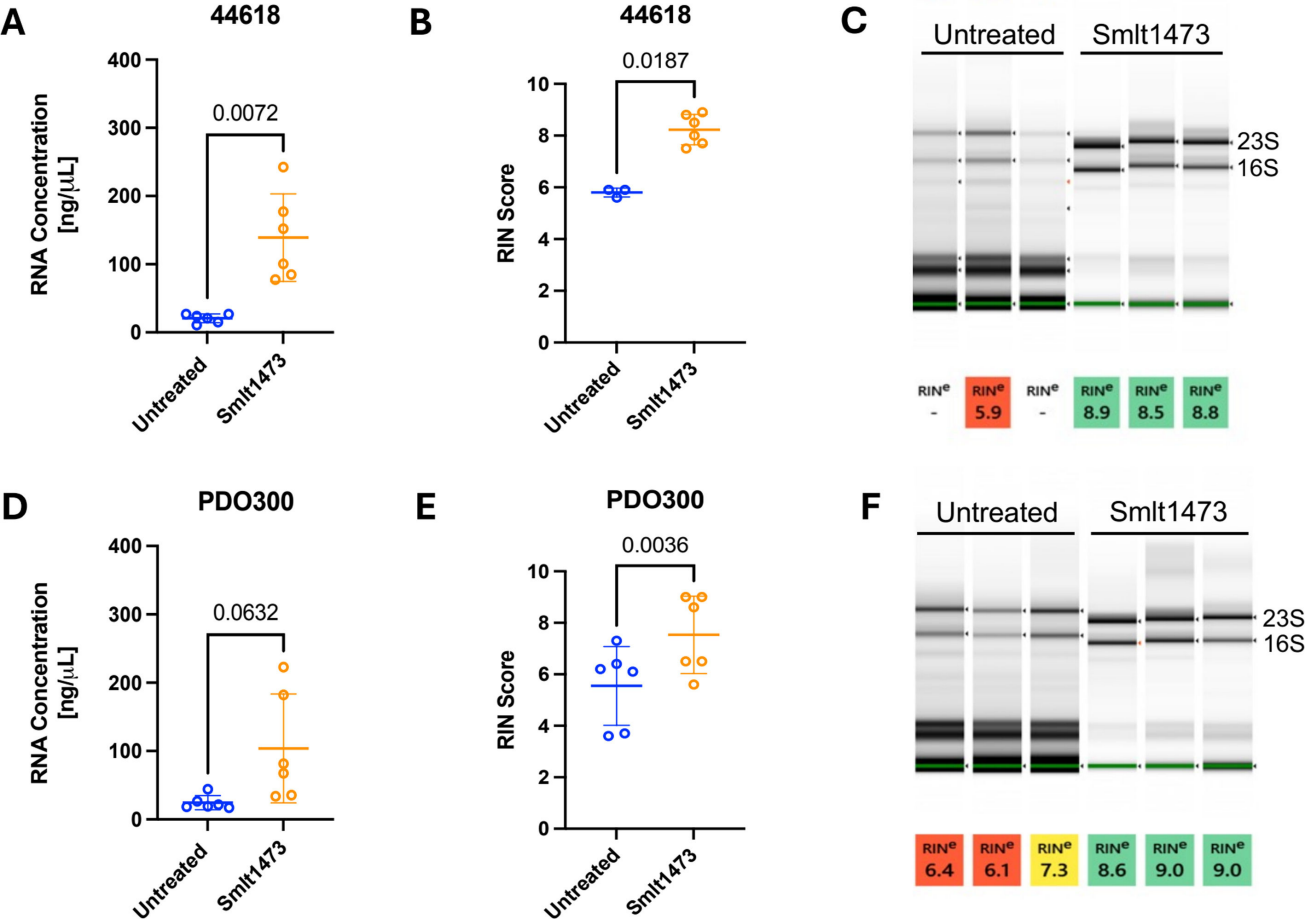
Addition of Smlt1473 to the RNA isolation process improves RNA quality and quantity for mucoid *P. aeruginosa* clinical isolate UVA 44618 and reference isolate PDO300. After the mucoid phenotype was established from incubation on LB agar plates at 37°C for 24 h, plate contents were collected. RNA was extracted using the Monarch Total RNA Miniprep Kit in either the presence or absence of 100 μg of Smlt1473. After extraction, RNA concentration was measured using the Qubit RNA BR Assay Kit. (**A**) Data show that addition of Smlt1473 significantly improves RNA concentration for UVA 44618. (**B**) RNA quality was determined by The University of Virginia’s Genome Analysis and Technology Core. RIN score data clearly demonstrate that addition of Smlt1473 significantly improves RNA quality for UVA 44618 as compared to the untreated samples. (**C**) Qualitative results from the RNA ScreenTape assay show that RNA samples from Smlt1473 treated samples are cleaner overall and have more defined bands for 23S and 16S as compared to the untreated samples. (**D**) Data show that while more variation is present for PDO300, the trend still suggests that addition of Smlt1473 improves RNA quantity. (**E**) RIN scores were determined by The University of Virginia’s Genome Analysis and Technology Core, and data show that addition of Smlt1473 during RNA isolation improves RNA quality for PDO300. (**F**) Qualitative results from RNA TapeStation show that RNA from PDO300 that was pre-treated with Smlt1473 is cleaner and has more defined bands at 23S and 16S as compared to untreated samples. The blue circles correspond to untreated samples and the orange circles correspond to enzyme treated samples. The results shown are means and standard deviations, and statistical analysis was performed using a two-tailed paired parametric *t*-test.

For the clinical isolate UVA44618, the presence of Smlt1473 significantly improved RNA yields obtained from the isolation procedure as shown in Fig. 2A by a significant increase in RNA concentration. The average RNA concentration for UVA44618 isolated without Smlt1473 was 20.7 ng/μL, while the average concentration with Smlt1473 was 139 ng/μL. Additionally, Smlt1473 significantly improved RNA quality as represented by RNA Integrity Number (RIN) scores and band intensity for 23S and 16S (Fig. 2B and C). The RIN is a numerical value ranging from 1 to 10 that is given to samples as an indicator of RNA integrity, with 10 being the least degraded (37). The integrity of RNA is important to consider for downstream sample processing, such as RNA sequencing, as degraded RNA will lead to inaccurate results (1). Of note for UVA44618, no RIN score was reported after quality analysis for three out of the six untreated replicates. The lack of a RIN score can be a result of concentration issues (too high or too low), degradation, or contamination (38–40). All three of these were cases where Smlt1473 was not used, and interestingly, when those same samples were pre-processed with Smlt1473, RIN scores ranging between 7.5 and 8.9 were reported. Overall, the average RIN for UVA44618 in the presence of Smlt1473 was 8.2 as compared to an average of 5.8 without Smlt1473. These data indicate that use of Smlt1473 during the isolation process improves RNA quality and quantity for heavy exopolysaccharide-producing *P. aeruginosa* clinical isolate UVA44618.

Since UVA44618 is a clinical isolate of *P. aeruginosa*, we also chose to extract RNA in the presence and absence of Smlt1473 from the mucoid laboratory reference strain PDO300. For PDO300, there was some variability in RNA concentration when Smlt1473 was used, but the data still trend toward the claim that use of Smlt1473 during isolation leads to greater RNA abundance (Fig. 2D). The average RNA concentration for PDO300 in the absence of Smlt1473 was 24.5 ng/μL, as compared to 103.9 ng/μL when Smlt1473 was used for isolation. Additionally, the RIN scores for PDO300 were significantly improved when Smlt1473 was added to the isolation process (Fig. 2E and F). The average RIN for untreated samples was 5.5, while the average RIN for samples with Smlt1473 used during isolation was 7.5. Similar to UVA44618, it is apparent that Smlt1473 improves RNA quality and quantity for *P. aeruginosa* strain PDO300. In addition to these mucoid isolates, we also wanted to determine how the addition of Smlt1473 to the isolation process would affect RNA from non-mucoid bacteria.

### Smlt1473 does not negatively affect the non-mucoid *P. aeruginosa* strain PA14

**Fig 3:**
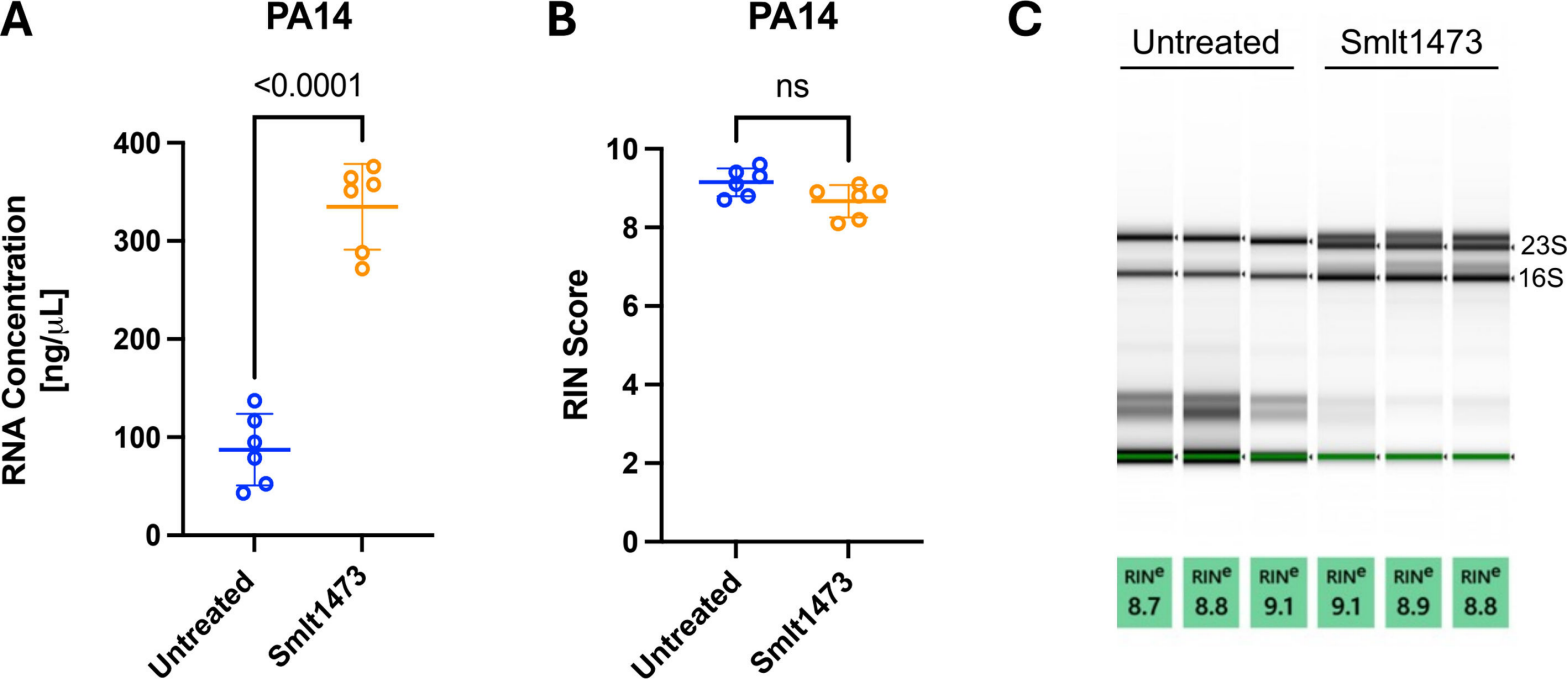
Smlt1473 improves RNA quantity for non-mucoid *P. aeruginosa* isolate PA14, but does not significantly affect quality. The same process to grow the bacteria and harvest the RNA was used for PA14 as was used for UVA 44618 and PDO300. (**A**) Resulting RNA concentrations from the Qubit RNA BR Assay show that Smlt1473 significantly improves the quantity of RNA extracted from PA14 samples as compared to those that were untreated. (**B and C**) Smlt1473 did not make a significant difference in RNA quality for non-mucoid isolate PA14. The blue circles correspond to untreated samples and the orange circles correspond to enzyme treated samples. The results shown are means and standard deviations, and statistical analysis was performed using a two-tailed paired parametric *t*-test.

To determine the effects of Smlt1473 on non-mucoid bacteria, we chose to isolate RNA from *P. aeruginosa* reference strain PA14. With the addition of 100 μg of Smlt1473 during the isolation process, RNA concentration was significantly improved for PA14 samples as compared to the samples isolated without Smlt1473 (Fig. 3A). The average concentration for RNA samples without Smlt1473 is 87.2 ng/μL, as compared to 334.8 ng/μL for RNA samples isolated with the addition of Smlt1473. While the RNA concentration was significantly improved for PA14 with the addition of Smlt1473 to the isolation process, the RNA integrity is not significantly different with the addition of Smlt1473 (Fig. 3B and C). The average RIN score without Smlt1473 was 9.1 versus 8.7 in the presence of Smlt1473. The original hypothesis for this work was that Smlt1473 would improve RNA quality for bacteria that are heavy mucoid EPS producers due to the enzyme’s ability to break down alginate and ultimately result in better release of RNA (34, 41). Reference strain PA14 was used to test whether Smlt1473 would negatively impact RNA isolation on visibly non-mucoid isolates. No significant difference in RNA quality was observed, suggesting that the enzyme does not adversely affect RNA isolation. While *P. aeruginosa* is well studied, we wanted to explore the effects of Smlt1473 on RNA isolation of other species to expand its application capabilities.

### Smlt1473 improves RNA isolation from the plant pathogen *Pseudomonas syringae*

**Fig 4:**
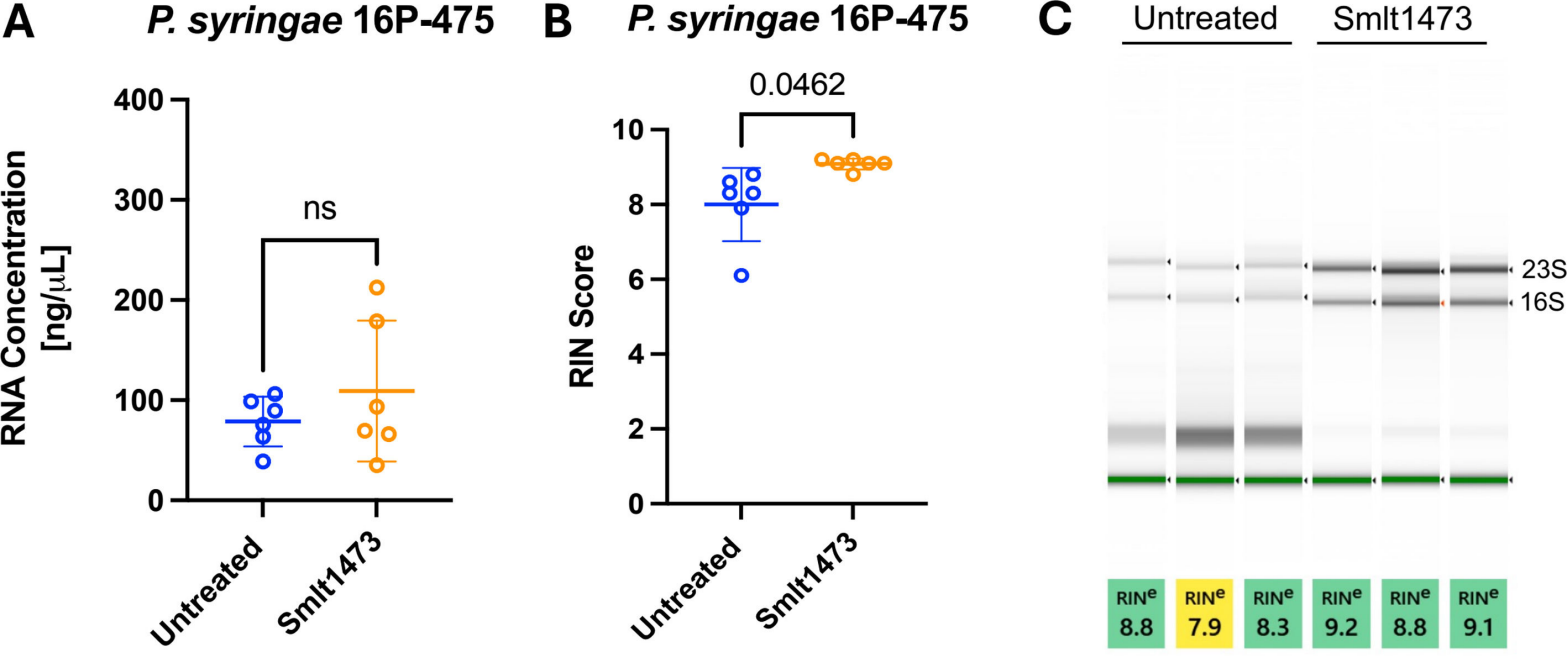
Smlt1473 improves RNA quality for *P. syringae*, but does not significantly affect quantity. After the mucoid phenotype was established from incubation on NSA plates at 28°C for 24 h, plate contents were collected. RNA was extracted using the Monarch Total RNA Miniprep Kit in either the presence or absence of 100 μg of Smlt1473. (**A**) RNA concentration was not significantly affected by the addition of Smlt1473 to the isolation process. (**B**) Addition of Smlt1473 to the isolation process significantly improved the RIN scores of RNA isolated from *P. syringae* 16P-475. (**C**) Qualitative data for RNA quality show that addition of Smlt1473 resulted in more defined bands for 23S and 16S. The blue circles correspond to untreated samples and the orange circles correspond to enzyme treated samples. The results shown are means and standard deviations, and statistical analysis was performed using a two-tailed paired parametric *t*-test.

To expand the application of our method beyond biomedical research, we chose to determine the effects of Smlt1473 on RNA isolation from a bacterium with a significant adverse impact in agriculture (42). *P. syringae* pv. tabaci is a plant pathogen that can cause wildfire and angular leaf spot in tobacco plants, and similar to other *Pseudomonas* species, the bacterium produces alginate (43–45). *P. syringae* strain 16P-475 (sensitive to streptomycin) was grown on nutrient sucrose agar (NSA) at 28°C for 24 h to establish its mucoid phenotype. As done for the *P. aeruginosa* strains, RNA was isolated in the presence and absence of 100 μg of Smlt1473. In the presence of Smlt1473, we observed variability in RNA concentration among samples, and overall, concentration was not significantly different when comparing isolations with and without enzyme (Fig. 4A). The average RNA concentration was 78.7 ng/μL without Smlt1473 and 109.3 ng/μL with Smlt1473. While RNA concentration was not significantly different for *P. syringae* 16P-475 in the presence of enzyme, the RNA quality was significantly improved, as shown by band intensity and RIN scores (Fig. 4B and C). These data show that by significantly improving RNA quality, Smlt1473 has a positive effect on RNA isolation from an agriculturally relevant species of *Pseudomonas*, consistent with results from clinical *P. aeruginosa* isolates where high levels of biofilm EPS are present.

### Use of Smlt1473 during RNA isolation improves gene assignment

**Fig 5:**
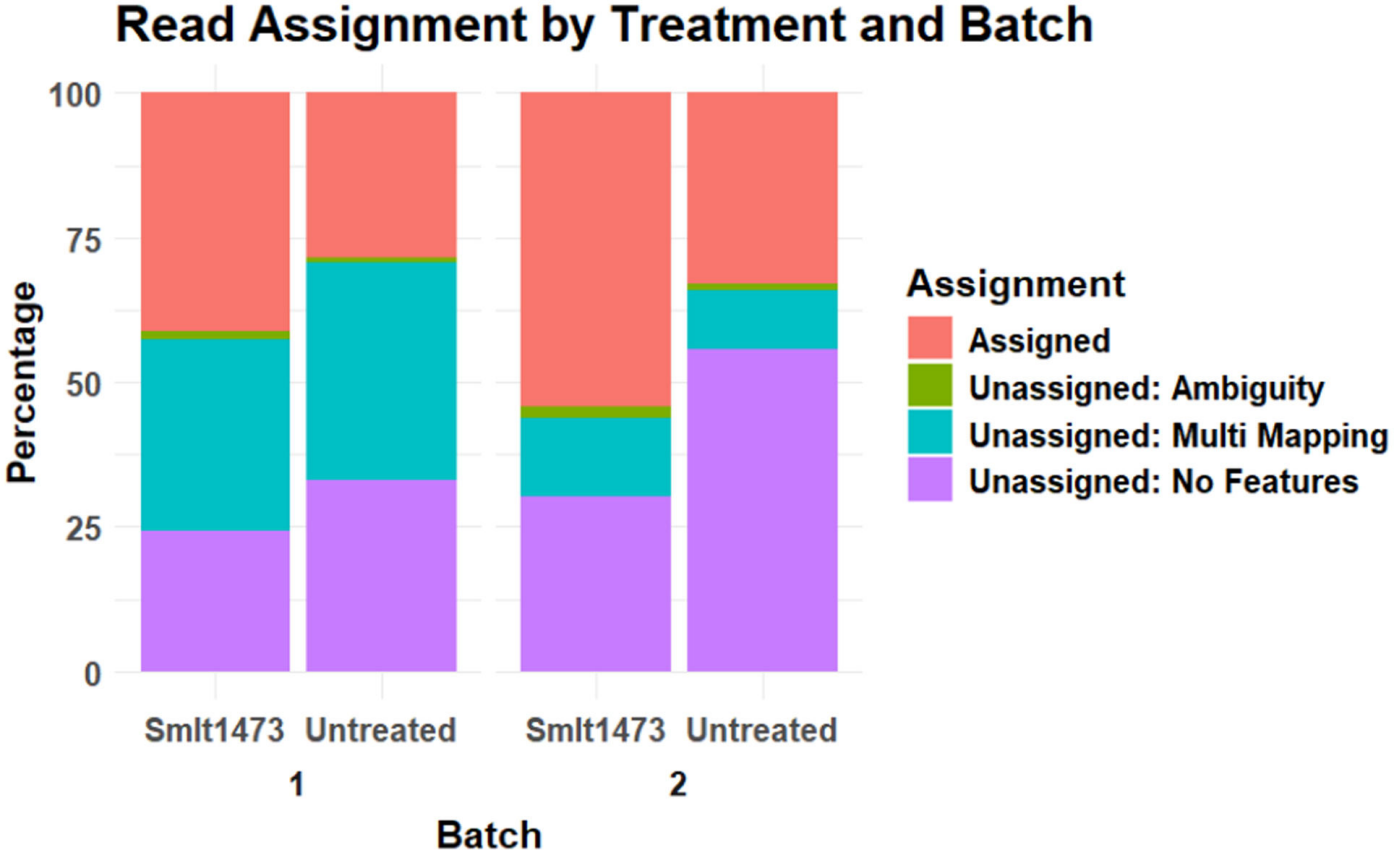
Smlt1473 improves RNA-seq read assignment and decreases the number of unassigned reads for *P. aeruginosa* isolate PA14. FastQC and MultiQC data show that for both batches of RNA isolation, Smlt1473 addition caused an increase in the proportion of reads that were able to be assigned during alignment. All RNA-seq data are from *P. aeruginosa* isolate PA14.

Since the goal of Smlt1473 is to enhance the RNA isolation process, we wanted to ensure that Smlt1473 could be beneficial to downstream RNA-seq analysis. We sequenced the transcriptome of PA14 (*n* = 6) with and without exposure to Smlt1473 during RNA isolation. One of the enzyme-treated isolates was excluded from further downstream analysis due to poor sequence quality and high variation from the other isolates in the batch. We found that the number of reads passing quality filtering was not affected by enzyme treatment (Fig. S1). This result is in accordance with the earlier finding that Smlt1473 does not change the quality of RNA isolated from PA14. On average, treatment with Smlt1473 increased the percent of assigned reads by 12%. This improvement was accompanied by a 23% decrease in unassigned reads due to no feature assignment (Fig. 5). We performed these isolations in two different batches. These results were consistent across each batch.

Additionally, we observed a modest increase in reads that mapped to multiple genomic locations in the enzyme-treated samples, likely reflecting the recovery of repetitive or highly expressed RNAs that are more accessible following biofilm matrix degradation. Collectively, the overall improvement in uniquely assigned reads indicates that Smlt1473 enhances the capture of informative RNA for downstream analysis.

### Addition of Smlt1473 to samples during the RNA isolation process does not alter gene expression

**Fig 6:**
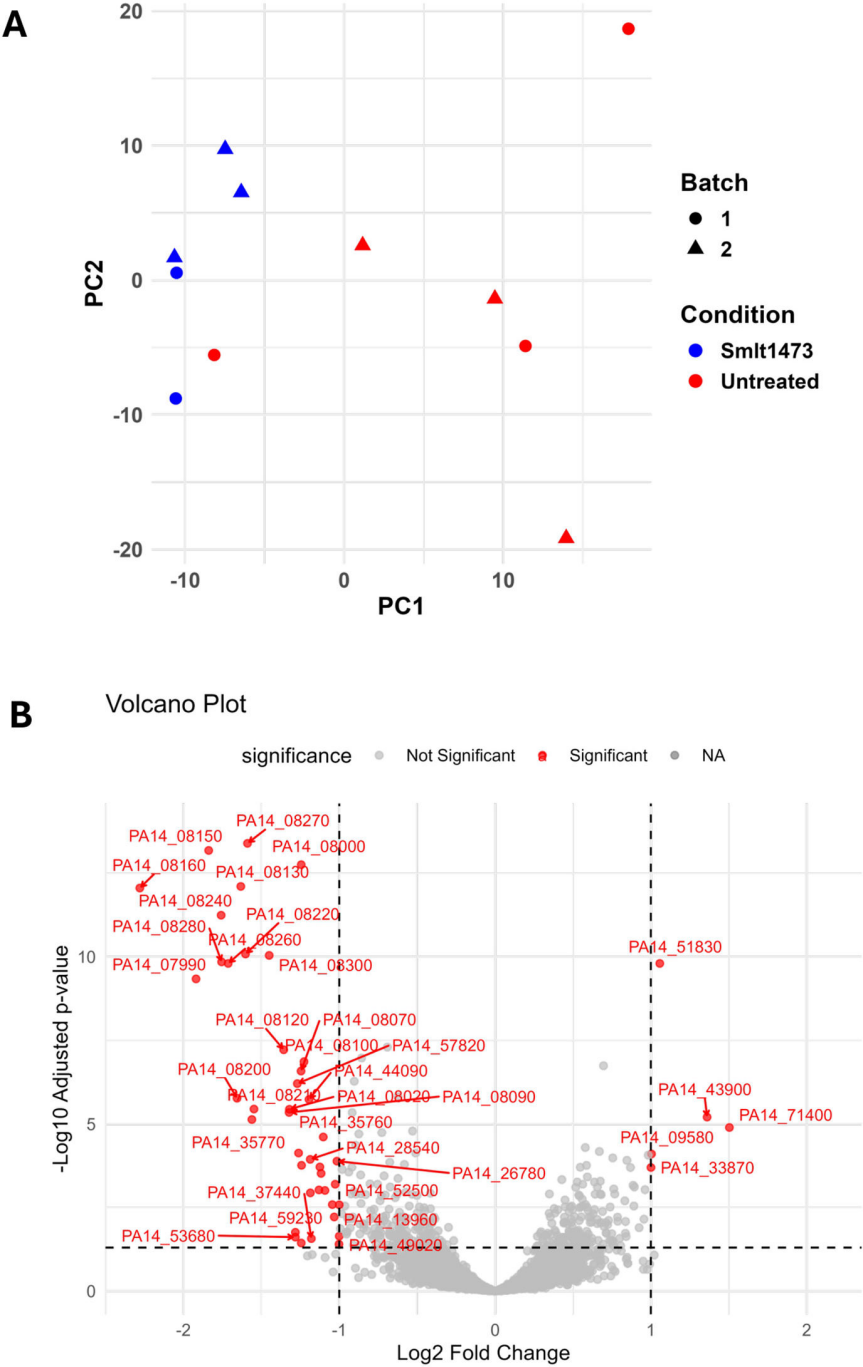
Smlt1473 introduces minor changes to overall gene expression for *P. aeruginosa* isolate PA14. (**A**) PCA was performed on the counts data from the RNA isolated from PA14 either untreated or exposed to Smlt1473. PCA was able to separate the treated from the untreated isolates, but the variation between isolates within the untreated group was much greater than that in the enzyme-treated group. (**B**) Differential expression between the untreated and enzyme-treated revealed some differentially expressed genes (DEGs). These genes were mostly hypothetical proteins with very few mappings to putative functions such as phage components and stress proteins. All RNA-seq data are from *P. aeruginosa* isolate PA14.

To identify whether the addition of Smlt1473 to the RNA isolation process affected global gene expression, we compared the expression profiles of the untreated samples to those of the enzyme-treated samples. We first plotted the overall expression profile for each sample using PCA (Fig. 6A). We found that most of the enzyme-treated samples were separated from the untreated samples along PC1. One of the untreated samples clustered with the enzyme-treated samples. Interestingly, the enzyme-treated group had much tighter overall clustering than the untreated groups, meaning that the enzyme treatment leads to a decrease in variance in overall gene expression between samples. A permutational multivariate analysis of variance was used to assess the effects of treatment condition on the distance matrix for each of these samples. The overall model was not statistically significant (*P* = 0.188), explaining approximately 24.3% of the variance in the distance matrix. The remaining 75% of the variance was attributable to residual variation. These results indicate that, when accounting for batch effects, enzyme treatment does not significantly contribute to the dissimilarity between samples.

When performing differential expression between the enzyme-treated and untreated groups, there were 46 total differentially expressed genes, with 5 genes significantly upregulated in the enzyme-treated condition and 41 genes downregulated in the enzyme-treated relative to the untreated (Fig. 6B) (log_2_FC > 1, padj < 0.05). None of the differentially expressed genes mapped to proteins with well-characterized functions, as most were mapped to hypothetical proteins. The most significantly upregulated gene mapped to a putative DNA-binding stress protein. Some genes, such as those between PA14_08240 and PA14_08280, were downregulated in the enzyme-treated condition and map to possible phage elements.

## DISCUSSION

In this study, we evaluated the ability of recombinantly produced polysaccharide lyase, Smlt1473, to improve RNA isolation from polysaccharide-rich bacteria, with a focus on both clinical and environmental *Pseudomonas* isolates. It has already been shown that Smlt1473 can inhibit and degrade alginate produced by *P. aeruginosa* clinical isolates (34). Additionally, it is proven that high polysaccharide content is a major hindrance to RNA isolation (20, 46). Therefore, we leveraged the ability of Smlt1473 to degrade alginate polysaccharides for improved RNA isolation from EPS-heavy *Pseudomonas* species.

For the use of our method, Smlt1473 was easily integrated into the protocol for a commercially available RNA isolation kit from New England Biolabs. Addition of lysozyme to the sample is a typical step early in the RNA isolation process, and we simply added Smlt1473 with lysozyme at this step (Fig. 1). Both solutions were added at the same time, and there was no additional incubation used, displaying ease of integration into the standard kit protocol. Our results show that the addition of 100 μg of Smlt1473 to the RNA isolation process improves both quantity and quality for mucoid EPS-producing *P. aeruginosa* isolates UVA44618 and PDO300 (Fig. 2). A previous study has shown that the composition of EPS for UVA44618 and PDO300 is predominantly alginate (34). Since Smlt1473 is known to degrade alginate and the primary EPS composition for these two mucoid *P. aeruginosa* isolates is alginate, during processing Smlt1473 breaks up the EPS allowing for more efficient cell lysis, release of RNA, and ultimately increased RNA yield (34, 41, 47). Based on the literature, polysaccharide content can also affect the purity of RNA by co-precipitating with it or by decreasing DNase I activity, resulting in gDNA contamination in the final sample (4). Therefore, improved RNA quality as shown by RIN scores, in the conditions where Smlt1473 was added is consistent with our expectation that removal of EPS enhances RNA recovery and quality. RNA quality and quantity are important for downstream analysis of the RNA by using techniques such as RNA-seq, RT-qPCR, and microarrays (3, 48, 49). For example, commercialized RNA-seq services often recommend 10–100 ng for short reads, 300–500 ng for long reads, and a RIN > 6 for best sequencing results (50, 51). Our method of enzymatic pre-processing to degrade polysaccharides present in the sample improves RNA concentrations and RIN scores to be within the ranges accepted for downstream processing. Overall, these data show that enzymatic pre-processing of mucoid *P. aeruginosa* isolates with Smlt1473 significantly improves RNA quantity and quality.

In addition to the mucoid isolates that produce significant amounts of alginate, we explored the use of our pre-processing method for strain PA14, a commonly used non-mucoid isolate of *P. aeruginosa*. PA14 produces a biofilm matrix composed of EPS, extracellular DNA, and proteins (52–54). When Smlt1473 was used in the process of RNA isolation for PA14, RNA yield significantly increased as compared to samples that were not pre-processed with the enzyme (Fig. 3A). This result could suggest that Smlt1473 is able to break up PA14 biofilm matrix or other cellular components (e.g., lipopolysaccharides), allowing lysozyme to have better access to the cells which could improve cell lysis and RNA release. Experiments beyond this study will be necessary to validate the activity of Smlt1473 against other components for improvements in RNA isolation.

Interestingly, even though Smlt1473 pre-processing improved RNA quantity, we did not observe an improvement in RNA quality for RNA isolated from PA14 (albeit the quality was already quite high) (Fig. 3B and C). Though there was not a significant improvement in RNA quality when comparing samples treated with the enzyme versus those that were not, in all cases, RIN scores for the PA14 isolate are acceptable for downstream processing (Fig. 3B and C). Isolating RNA from this non-mucoid strain clearly does not pose the same challenge to RNA isolation that mucoid strains do. Therefore, this isolate was used as a control to show that enzyme pre-processing is not detrimental to RNA isolation. This result also suggests that not all polysaccharide types may affect RNA isolation similarly, and that the enzymatic pre-processing step is isolate dependent. To expand beyond medically relevant bacteria, we also evaluated our method for an agriculturally important bacterium, *P. syringae* 16P-475. Similar to the mucoid *P. aeruginosa* isolates, *P. syringae* EPS is composed of alginate, though it does contain other polysaccharides such as levan and cellulose that differentiate it (55). Since this *Pseudomonad* species has an EPS composed in part of alginate, and this particular isolate does present a mucoid phenotype similar to that of the *P. aeruginosa* isolates presented in this study, *P. syringae* 16P-475 is a good candidate to evaluate our method beyond *P. aeruginosa*. In the case of *P. syringae* 16P-475, we did not see a significant increase in RNA quantity when Smlt1473 was used (Fig. 4A), though variability in the concentration data for the enzymatic pre-processed samples for this isolate could explain this result. On the other hand, similar to the mucoid isolates, we did see a significant improvement in RNA quality (Fig. 4B and C). Since *P. syringae* produces alginate and we visibly observe a mucoid phenotype when it is grown on agar plates, improved RNA quality with enzyme pre-processing is consistent with the hypothesis that enzymatic degradation of polysaccharides can reduce contaminants and improve RNA quality.

An analysis of transcript abundance revealed that the mean normalized expression of DEGs did not differ significantly from the non-DEGs (Fig. S2). This suggests that the observed expression changes were not biased toward low-abundance transcripts, indicating that the detected differences are unlikely to be artifacts of low signal-to-noise ratios. The decreased expression of phage-associated transcripts in the enzyme-treated samples may result from the biofilm-degrading activity of Smlt1473, which could preferentially recover RNA from intact, metabolically active cells while reducing the contribution of RNA from lysed cells or extracellular phage-containing material (34). It has also been shown that certain phage transcripts bind with proteins that protect them from degradation as they leave the nucleus (56). It is possible that Smlt1473 may interact with these proteins, resulting in a disruption of these long phage operon transcripts and a decreased expression of the phage tail operon. Further work is necessary to determine the specific mechanism by which this phage tail operon is downregulated in the enzyme-treated group.

Upregulation of one DNA-binding stress protein may reflect mild cellular stress induced during RNA isolation, as biofilm degradation transiently exposes cells to mechanical or chemical stress. However, only a single stress-response gene was differentially expressed, suggesting that this is unlikely to represent a true physiological stress response due to the addition of Smlt1473. Additionally, Smlt1473 was added after RNA stabilization, so it is unlikely that a full stress response was activated after the RNA was stabilized. Interestingly, changes in hypothetical protein expression may reflect enhanced recovery of RNA that is normally lost in the processing of untreated biofilms, revealing additional details regarding the physiological state of the cell. Overall, these results indicate that while Smlt1473 treatment slightly affects the expression of a small set of genes, the changes are limited and largely involve hypothetical proteins or potential phage-associated genes (Fig. 6B). Importantly, the tight clustering of enzyme-treated samples and the relatively small number of differentially expressed genes suggest that Smlt1473 does not introduce widespread transcriptional changes. This result supports the conclusion that the enzyme primarily improves RNA isolation efficiency and gene assignment without substantially altering the underlying gene expression profile of PA14 isolates. Although there were some differentially expressed genes, many are phage genes and unspecified proteins, meaning that these changes are unlikely to reflect biologically meaningful alterations in core cellular pathways. Taken together, these observations indicate that Smlt1473 is an effective tool for enhancing RNA-seq data quality by reducing technical variability and improving read assignment, while maintaining the integrity of the global transcriptional landscape.

The results presented in this manuscript highlight the advantages of pre-processing samples with recombinant polysaccharide lyase, Smlt1473, during the RNA isolation process. In the cases presented, we observe an increase in RNA quality and/or quantity in the presence of Smlt1473 compared to cases where the enzyme was not used, making RNA more suitable for downstream analysis. The amount of Smlt1473 used in this study was 100 μg; however, this amount could be tailored for specific use cases or isolates. Additionally, depending on the sample type and polysaccharide content, different incubation times with the enzyme alone could also be beneficial before proceeding with cell lysis and the rest of the protocol. While this paper focuses on *Pseudomonas* species because of their clinical and environmental relevance as initial proof-of-concept, in the future, we plan to extend this approach to other important environmental and clinical pathogens that produce EPS.

## MATERIALS AND METHODS

### Protein expression and purification

An *Escherichia coli* codon-optimized nucleotide sequence of Smlt1473 (GenBank accession number CAQ45011), cloned into pET-28a(+) using NcoI/XhoI restriction sites (GenScript), was used. The coding sequence contains a C-terminal hexahistidine tag suitable for protein purification via immobilized metal affinity chromatography (IMAC). The plasmid was transformed into *E. coli* BL21 cells for protein expression. Purification was done using previously described methods with a few modifications to improve protein yield (41). After induction with 1 mM isopropyl β-D-1-thiogalactopyranoside for 16–18 h at 20°C, cells were harvested by centrifugation and resuspended in 2× lysis buffer made from a 1 L 5× stock solution (250 mL glycerol 99%, 22.2 g Tris HCl, 13.3 g Tris Base, 29.2 g NaCl, 1.7 g imidazole, adjusted to pH 8, and then sterilized). The mixture was frozen at −80°C for 30 min, followed by thawing at 37°C for 30 min. Once thawed, the cell mixture was sonicated at 20% amplitude for 20 min with 20 s on/off intervals. After sonication, the supernatant containing protein was collected and purified via IMAC. Protein was separated using 10, 20, 30, 75, 100, and 250 mM imidazole elutions. Samples were analyzed via SDS-PAGE to determine purity, followed by dialysis of pure samples in 20 mM sodium phosphate buffer (pH 8) overnight at 4°C. Protein aliquots were stored at −20°C for future use. Prior to each experiment, protein concentration was determined by measuring absorbance at 280 nm and using a calculated extinction coefficient of 74,495 M^−1^ cm^−1^.

### Bacterial strains, culture media, and growth conditions

In this study, three strains of *P. aeruginosa* were used. Clinical isolate UVA44618 is from a collection of isolates from the University of Virginia hospital acquired from February 2019 to February 2020, as described in a publication by Dunphy et al. (35). The isolation site for UVA44618 was the lung/trachea of a patient with cystic fibrosis, and this isolate displays the mucoid phenotype which is defined by the overproduction of exopolysaccharide alginate (33). Additionally, this study used PDO300, which was kindly provided by Dr. Joanna Goldberg at Emory University School of Medicine. PDO300 is a defined isogenic derivative of PAO1 that was engineered to have the *mucA22* allele, allowing it to constitutively overproduce the alginate exopolysaccharide and therefore also displays the mucoid phenotype (36). Laboratory reference strain PA14 was also used to explore a strain classified as non-mucoid. *Pseudomonas syringae* pv. tabaci (Pst) strain 16P-475 (sensitive to streptomycin) was kindly provided by Ed Dixon in Plant Pathology at the University of Kentucky. Similar to the *P. aeruginosa* strains used in this study, *P. syringae* 16P-475 also produces alginate and has a viscous appearance representative of the mucoid phenotype (57).

For both *Pseudomonas* species, the mucoid phenotype is visible and most prominent on agar plates, and therefore bacterial strains were grown on plates rather than in liquid media for the experiments in this study. All three of the *P. aeruginosa* strains were streaked on LB agar and left to incubate at 37°C for 24 h. For storage of *P. aeruginosa*, bacterial cells were resuspended in LB media, mixed with glycerol for a final concentration of 25%, and frozen stocks were stored at −80°C. To acquire the mucoid phenotype of *P. syringae*, bacteria were grown on NSA at 28°C for 24 h. For storage of *P. syringae*, bacterial cells were resuspended in King’s B media, mixed with glycerol for a final concentration of 25%, and frozen stocks were stored at −80°C.

### RNA isolation

Various bacterial strains were streaked from frozen stock onto agar plates. *P. aeruginosa* strains grew on LB agar, while the *P. syringae* strain grew on NSA. *P. aeruginosa* plates were grown at 37°C for 24 h, and *P. syringae* plates were grown at 28°C for 24 h. Plate contents were collected using 10 mL of 0.85% NaCl, followed by vortexing to homogenize the solution. Next, 300 μL of culture was added to 300 μL of a cold 1:1 ethanol:acetone solution to stabilize RNA and mixed. The mixture was centrifuged at 10,000 rpm for 5 min, and then the supernatant was discarded. For each isolate, there were three aliquots that received enzyme treatment and three aliquots from the same three main culture tubes that did not. This was repeated across two separate days for a total of six samples per condition per isolate. For those that did not receive enzyme, 25 μL of 4 mg/mL lysozyme (Affymetrix) was added. For those receiving enzyme treatment, 25 μL of 4 mg/mL lysozyme was added, in addition to 100 μg of Smlt1473. Pilot studies explored the addition of Smlt1473 at various amounts from 100 to 300 μg, and 100 μg of Smlt1473 was chosen for use in this study. These samples were mixed, and then 800 μL of lysis buffer from the Monarch Total RNA Miniprep Kit (now NEB #T2110) was added. For the remainder of isolation, the protocol from the Monarch Total RNA Miniprep Kit was followed. An overview of the method is shown in Fig. 1. Of note, the DNase I included in the kit gives the best results and should not be substituted with other DNase I. RNA was eluted using 100 μL of nuclease-free water and stored at −80°C. RNA concentrations were measured using the Qubit RNA BR Assay Kit, and RNA quality was determined by The University of Virginia’s Genome Analysis and Technology Core, RRID:SCR_018883 using the RNA ScreenTape assay.

### RNA sequencing

We chose to use PA14 for RNA-seq because it is a widely used reference strain and the genome is well annotated (58, 59), thereby providing more complete mapping to genes and easier analysis (as compared to the clinical isolates which require more extensive *de novo* transcriptomic mapping). After RNA isolation was performed, samples were stored at −80°C to preserve stability. For analysis, 20 μL of sample with a concentration > 50 ng/μL and RIN > 6 was aliquoted and sent frozen on dry ice to SeqCenter in Pittsburgh, PA for RNA-seq analysis (60). Samples were DNase-treated with Invitrogen DNase (RNase-free). Library preparation was performed using Illumina’s Stranded Total RNA Prep Ligation with Ribo-Zero Plus Kit and 10 bp unique dual indices. Sequencing was done on a NovaSeq X Plus, producing paired-end 150 bp reads. Demultiplexing, quality control, and adapter trimming were performed with bcl-convert (version 4.2.4) (61). Further trimming was performed with fastp followed by quality checking with fastqc (62, 63). Reads were aligned to the UCBPP-PA14 genome using the STAR aligner on default settings, and counts were tabulated using FeatureCounts (64, 65). Differential expression analysis was performed with DESeq2. All code for processing reads and data analysis is posted on GitHub at [https://github.com/csbl/SMLT_sequencing].

### Statistical analysis

For each isolate, there were three aliquots that received enzyme treatment and three aliquots from the same three main culture tubes that did not. This was repeated across two separate days for a total of six samples per condition per isolate. Statistical analysis was performed using GraphPad Prism version 10.2.1. Values in all graphs are expressed as mean with standard deviation. Comparisons were done using a two-tailed paired parametric *t*-test. *P* values are provided on the respective data figures in the manuscript.

## SUPPLEMENTAL MATERIAL

Supplemental figures spectrum.03077-25-s0001.docx Fig. S1 and S2.

ASM does not own the copyrights to Supplemental Material that may be linked to, or accessed through, an article. The authors have granted ASM a non-exclusive, world-wide license to publish the Supplemental Material files. Please contact the corresponding author directly for reuse.
