# Supplementary material for: Enzyme-enhanced RNA isolation from biofilm-producing bacteria
Source: Microbiol Spectr. 2026 Feb 27;14(4):e03077-25. doi: 10.1128/spectrum.03077-25 (PMC13055276; doi:10.1128/spectrum.03077-25)
Supplement: Supplemental figures — Fig. S1 and S2. [file spectrum.03077-25-s0001.docx]

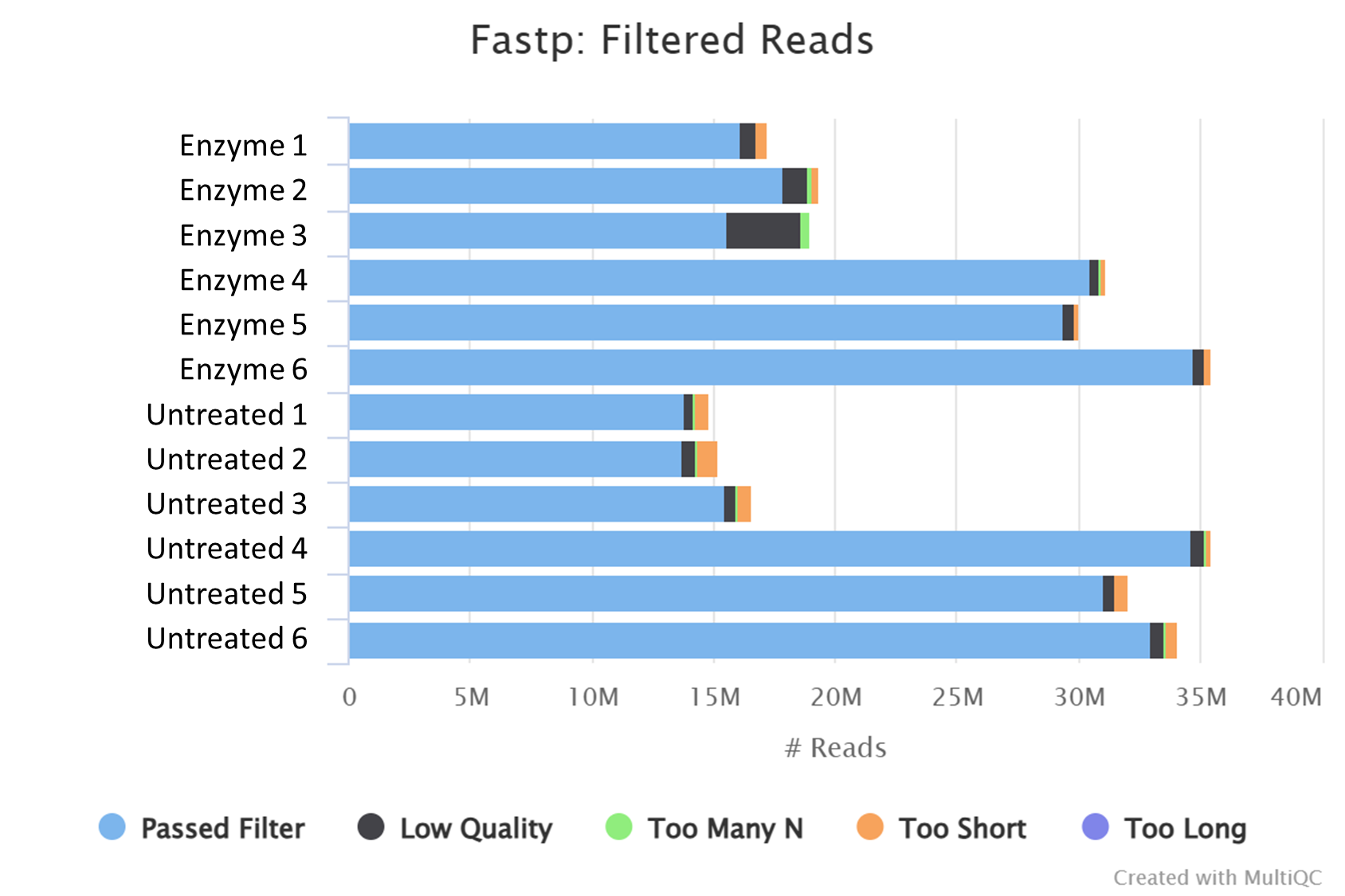


**Figure S1.** Quantification of reads that passed the Fastp quality filtering. Replicates 1-3 for the enzyme and untreated were from the first batch and 4-6 from the second batch. Within each batch, there is minimal variation in the number of reads and reads that passed the filtering.


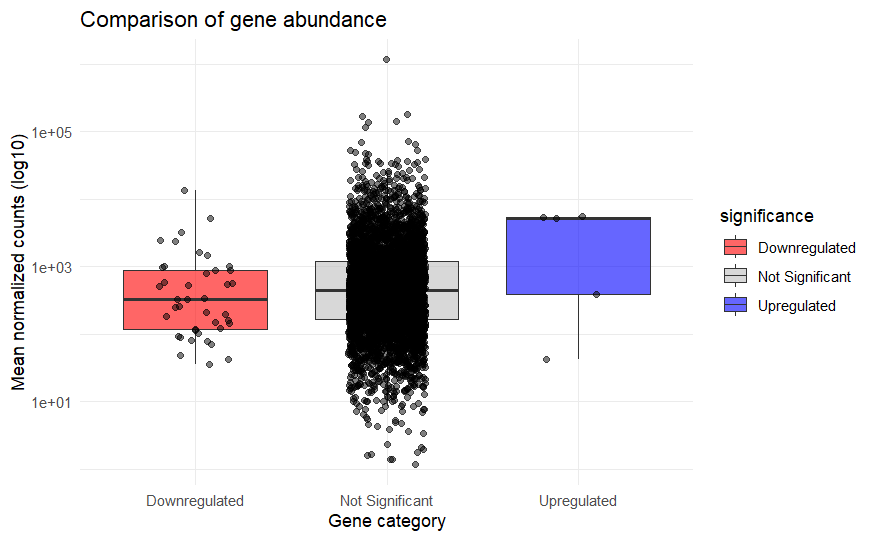


**Figure S2.** Comparison of mean transcript abundances across genes that were significantly differentially expressed and those that were unchanged. A Wilcoxon test was run to see if there was any difference between the groups but there was no significance with the cutoff of p<0.05.
